# Tropomyosin isoform Tpm2.1 regulates collective and amoeboid cell migration and cell aggregation in breast epithelial cells

**DOI:** 10.18632/oncotarget.19182

**Published:** 2017-07-12

**Authors:** HyeRim Shin, Dayoung Kim, David M. Helfman

**Affiliations:** ^1^ Department of Biological Sciences, Korea Advanced Institute of Science and Technology, Daejeon, Republic of Korea

**Keywords:** collective cell migration, amoeboid migration, cell aggregation, AXL receptor tyrosine kinase, metastasis

## Abstract

Metastasis dissemination is the result of various processes including cell migration and cell aggregation. These processes involve alterations in the expression and organization of cytoskeletal and adhesion proteins in tumor cells. Alterations in actin filaments and their binding partners are known to be key players in metastasis. Downregulation of specific tropomyosin (Tpm) isoforms is a common characteristic of transformed cells. In this study, we examined the role of Tpm2.1 in non-transformed MCF10A breast epithelial cells in cell migration and cell aggregation, because this isoform is downregulated in primary and metastatic breast cancer as well as various breast cancer cell lines. Downregulation of Tpm2.1 using siRNA or shRNA resulted in retardation of collective cell migration but increase in single cell migration and invasion. Loss of Tpm2.1 is associated with enhanced actomyosin contractility and increased expression of E-cadherin and β-catenin. Furthermore, inhibition of Rho-associated kinase (ROCK) recovered collective cell migration in Tpm2.1-silenced cells. We also found that Tpm2.1-silenced cells formed more compacted spheroids and exhibited faster cell motility when spheroids were re-plated on 2D surfaces coated with fibronectin and collagen. When Tpm2.1 was downregulated, we observed a decrease in the level of AXL receptor tyrosine kinase, which may explain the increased levels of E-cadherin and β-catenin. These studies demonstrate that Tpm2.1 functions as an important regulator of cell migration and cell aggregation in breast epithelial cells. These findings suggest that downregulation of Tpm2.1 may play a critical role during tumor progression by facilitating the metastatic potential of tumor cells.

## INTRODUCTION

Metastasis is the major cause of cancer-associated mortality. Metastasis dissemination is due to various processes including cell migration and cell aggregation. Metastatic cells are known for their ability to migrate and invade into the microvasculature of the lymph and blood systems, survive from detachment before entering distant tissues and adapting to the foreign microenvironment for colonization. During invasion, cells exhibit different types of motility including amoeboid, collective, and mesenchymal or single cell migration. After intravasation, cells travel through the circulation before entering secondary sites, where they extravasate to the distant organ [1–3]. In order to understand and prevent metastasis, a detailed understanding of the mechanisms underlying metastasis is essential.

Disruption of cell-cell contacts, cell polarity, and alterations of the actin based cytoskeleton are features of transformed cells. In normal epithelial cells, the actin cytoskeleton associates with adherens and tight junctions [4]. Alterations in the organization of the cytoskeleton and deregulation in cytoskeletal dynamics contributes to phenotypic properties of cancer cells [3, 5, 6]. Actin filament bundles, also called stress fibers, can be divided into three categories based on their localization namely dorsal, ventral and transverse arc [6, 7]. These fibers are involved in mechanotransduction of cells by anchoring cadherins for cell-cell adhesion and focal adhesions which associate with the extracellular matrix (ECM) [8, 9]. Deregulation of these adhesion complexes are associated with abnormal signaling pathways in transformed cells [10, 11].

Tropomyosins (Tpms) are a family of actin filament binding proteins. There are 6 isoforms encoded by 4 genes expressed in breast epithelial cells. Tpm, is a dimeric coiled coil protein, and different isoforms are associated with different structures [12]. Tpm participates in the regulation of various cellular functions including cell motility, adhesion, signaling, vesicle transport and actomyosin contractility [12, 13]. Normal breast epithelial cells express three high molecular weight (HMW) proteins of 284 amino acids in length, termed Tpm2.1, Tpm1.7 and Tpm4.1, encoded by the TPM2, TPM1 and TPM4 genes, respectively, and three low molecular weight isoforms (LMW) of 248 amino acids in length, termed Tpm4.2, Tpm3.1, and Tpm1.9 encoded by TPM4, TPM3 and TPM1 genes, respectively [14, 15]. Previous studies have reported that downregulation of high molecular weight Tpm isoforms in epithelial cells is associated with the transformed phenotype and introduction of specific Tpm isoforms can reverse the properties of the transformed phenotype [16]. Additionally, Tpm isoforms appeared to have nonredundant functions in non-muscle cells. Thus, downregulation of specific Tpm isoforms may contribute to different properties of transformed cells [16–20].

In this study, we interrogated the role of Tpm2.1 in breast epithelial cells and how loss of Tpm2.1 expression contributes to tumor progression. These studies demonstrate that Tpm2.1 functions as an important regulator of cell migration and cell aggregation. Our data suggests that decreased expression of Tpm2.1 may play a pivotal role during tumor progression by facilitating the metastatic potential of tumor cells via its effects on cell migration and cell aggregation.

## RESULTS

### Tpm2.1 is downregulated in breast cancer and decreased expression of Tpm2.1 in MCF10A retards wound healing migration

**Figure 1:**
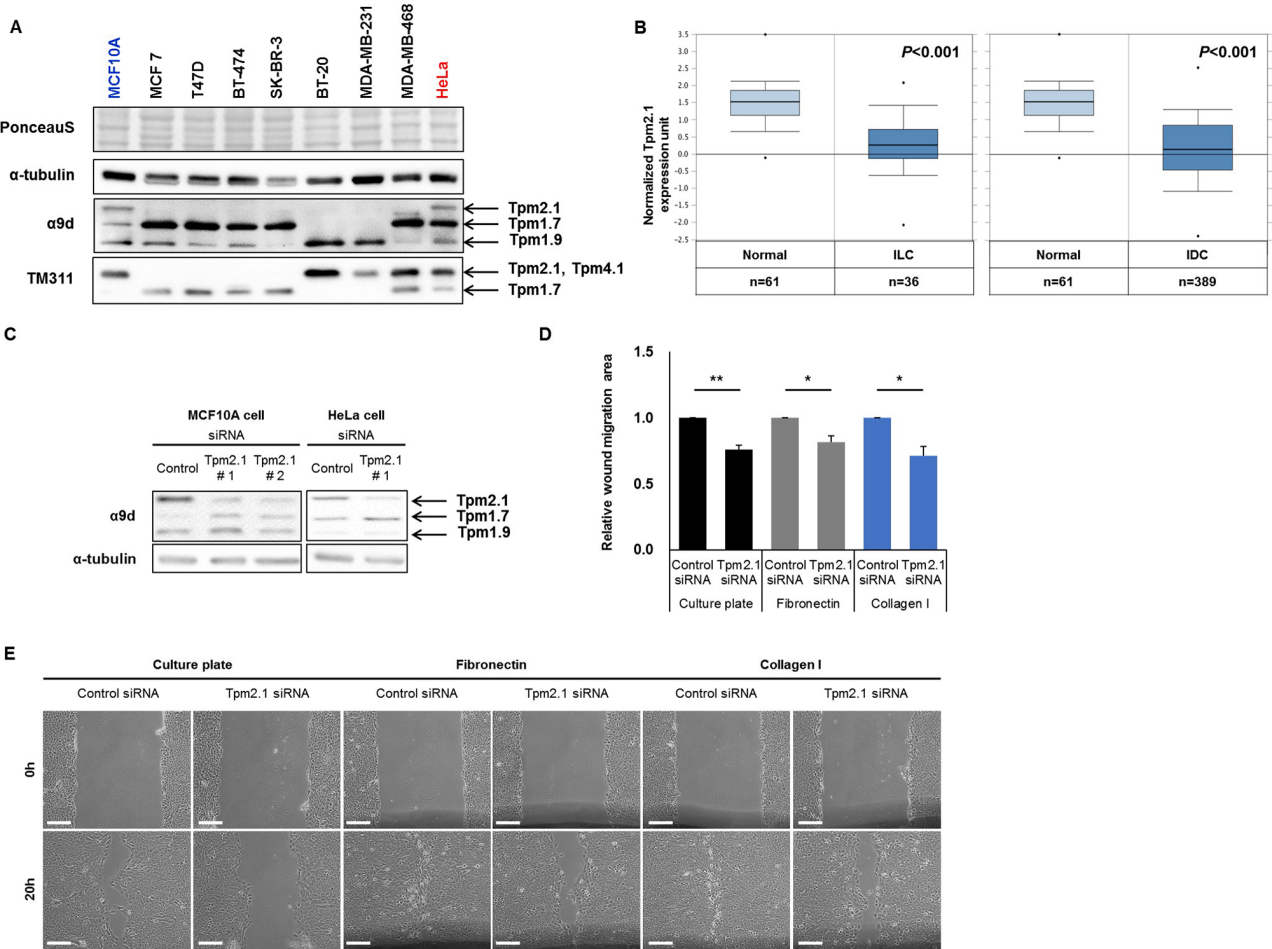
Tpm2.1 is downregulated in breast cancer and Tpm2.1-silencing in MCF10A retards wound healing migration independent of 2D substrates (**A**) Expression of Tpm isoforms were detected in breast epithelial (MCF10A), breast cancer (MCF7, T47D, BT-474, SK-BR-3, BT-20, MDA-MB-231 and MDA-MB-468) and cervical cancer (HeLa) cell lines against α9d and TM311 antibody. (**B**) Oncomine (http://www.omcomine.org) data of Tpm2.1 in normal vs metastatic cancer patients. Tested samples are mentioned below the graph; normal versus invasive lobular breast carcinoma (ILC), *P*-value: 2.26 × 10^-10^; fold change: −2.256 and normal versus invasive ductal breast carcinoma (IDC), *P*-value: 4.57 × 10^-25^; fold change: −2.496. (**C**) MCF10A cells were treated with siRNA against Tpm2.1 for 72 hours and silencing was detected using immunoblotting. α-tubulin was used as a loading control. Error bars indicated means ± s.e.m; *P < 0.05, **P < 0.01 as compared with control, Student's *t*-test. (**D**–**E**) More than three independent experiments of siRNA-treated cells were grown to confluent monolayer on different substrates and quantified by wound closure area by Image J (Scale bar: 100 μm).

We first compared the expression of Tpm2.1 in different breast cancer epithelial cell lines. Tpm2.1 was detected only in non-transformed breast epithelial cells and undetectable in various breast cancer cell lines including MCF7, T47D, BT-474, SK-BR-3, BT-20, MDA-MB-231, and MDA-MB-468 (Figure 1A). This is in agreement with previous studies showing Tpm2.1 is downregulated in breast cancer cell lines [15, 21, 22]. To further gain insight into the involvement of Tpm2.1 in breast cancer patients, we analyzed the Oncomine gene expression database comparing normal versus breast cancer. Results from 593 available samples showed downregulation of Tpm2.1 levels in invasive lobular and ductal breast carcinoma tissues (Figure 1B). Thus, downregulation of Tpm2.1 is associated with breast cancer progression and metastatic disease.

**Figure 2:**
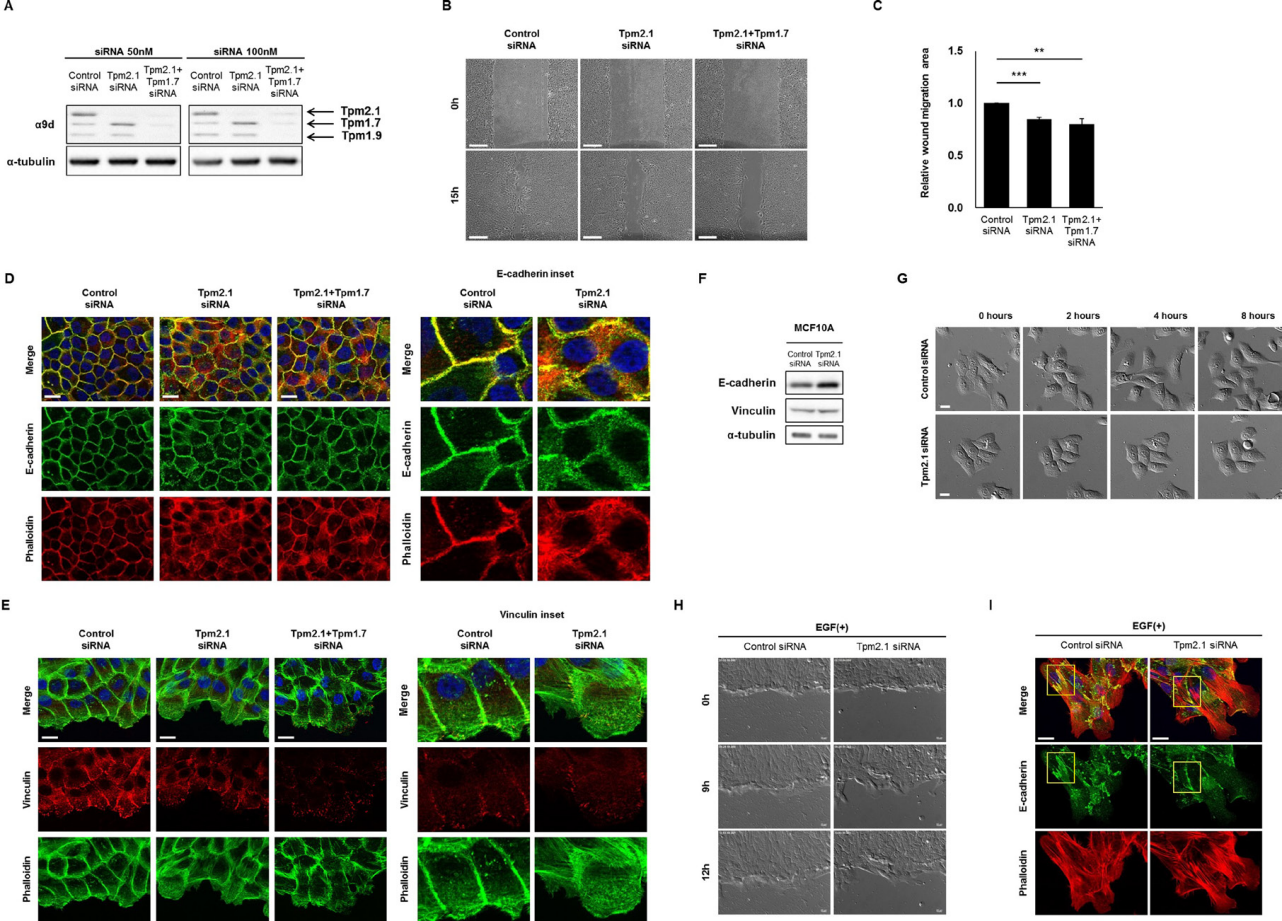
Downregulation of Tpm2.1 retards collective cell migration (**A**) Protein expression of Tpms were detected after 50, 100 nM RNAi treatment in MCF10A cells. (**B**) Collective cell migration was monitored at 0 and 15 hours and (**C**) quantified the area of wound closure using Image J (Scale bar: 100 μm). More than three independent experiments were performed (means ± s.e.m; **P < 0.01, ***P < 0.001; Student's *t-test*). (**D**–**E**) RNAi treated cells were stained against E-cadherin and vinculin together with phalloidin and DAPI under confluent cell state, and at the cell edge of wound, in confluent monolayer. Magnified images are represented for detailed image (Scale bar: 20 μm). (**F**) Immunoblotting against E-cadherin and vinculin after RNAi treatment in MCF10A cells. α-tubulin was used as a loading control. (**G**) MCF10A cell clusters were cultured for 24 hours under serum and growth factor starved condition followed by 100 ng/ml EGF treatment to monitor cell scatter (Scale bar: 20 μm). (**H**) MCF10A cells cultured for 24 hours under serum and growth factor starved condition were wounded and monitored for 12 hours after 100 ng/ml EGF treatment (Scale bar: 20 μm). (**I**) EGF treated cells, cultured for 24 hours were stained against E-cadherin (yellow box) with phalloidin and DAPI (Scale bar: 20 μm).

To elucidate how downregulation of Tpm2.1 can contribute to breast cancer progression we interrogated the role of Tpm2.1 in non-transformed MCF10A breast epithelial cells. Previously, researchers reported that loss of HMW Tpm is involved in enhanced cell migration and invasion [16, 23]. In order to determine if loss of Tpm2.1 is associated with alterations in cell motility we performed gene knockdown using RNAi treatment in MCF10A cells. Both MCF10A and HeLa cell showed efficient knockdown of Tpm2.1 expression following treatment with siRNA. To eliminate the off-target effects of RNAi treatment, we used two different RNAi against Tpm2.1 and also tested in their effects in HeLa cells (Figure 1C). We first asked if loss of Tpm2.1 played a role in collective cell migration using wound healing assay. Cells were plated on uncoated culture plate, fibronectin or collagen, treated with siRNA and then wounded. We compared the rate of migration using different matrices because some studies reported that cells exhibit faster motility rates on fibronectin or collagen [24–26]. Surprisingly, downregulation of Tpm2.1 resulted in a decreased rate of wound closure, whether cells were plated on culture plates alone or culture plates coated with fibronectin or collagen I (Figure 1D and 1E). Curiously, we observed that siRNA treatment against Tpm2.1 resulted in upregulation of another HMW Tpm, namely Tpm1.7 (Figure 1C). One possible explanation for the slower rate of wound healing migration following downregulation Tpm2.1 was the increase level of Tpm1.7 also expressed in breast epithelial cells. To test this hypothesis we performed RNAi treatment against Tpm1.7 and Tpm2.1, parallel to Tpm2.1 RNAi treatment alone (Figure 2A). To our surprise, the upregulation of Tpm1.7 showed no affect in collective migration because siRNA against Tpm1.7 did not affect the slower migration following downregulation of Tpm2.1 (Figure 2B and 2C). Thus, downregulation of Tpm2.1 led to a slower rate of collective migration.

To further examine how Tpm2.1 depletion affects cell motility, we analyzed the organization of the actin cytoskeleton and cell adhesions following inhibition of Tpm2.1. In control cells, actin organization and E-cadherin exhibited well organized linear structures at cell-cell contacts in confluent cells, whereas Tpm2.1-depleted cells showed dispersed localization of E-cadherin around the cortical membrane and disruption of cortical actin filaments (Figure 2D). Furthermore, analysis of cells at the edge of wound during collective migration revealed the cortical actin alignment was largely disrupted and cells showed a decrease in the number and size of vinculin containing focal adhesions (Figure 2E). Immunoblotting data showed decreased expression of Tpm2.1 was associated with an increase in the levels of E-cadherin and β-catenin expression but no change in the level of vinculin expression (Figure 2F, Supplementary Figure 1). Hence, downregulation of Tpm2.1 disrupted cell-cell adhesions and cortical actin filaments.

Epithelial cell scatter is a useful *in vitro* model for the study of epithelial-to-mesenchymal transition (EMT) [27, 28]. We used this model to study MCF10A cell motility after Tpm2.1-silencing, followed by EGF treatment under serum and growth factors starved condition. Cells were grown into well-defined clusters in growth factor deprived media then treated with EGF. When control cells were treated with EGF, they showed disruption of cell contacts between neighboring cells and enhanced cell migration (Figure 2G). By contrast, Tpm2.1-silenced cells showed no scatter from the cell cluster following treatment with EGF (Figure 2G). We also examined the effects of EGF treatment on wound healing. Treatment of cells with EGF during wound healing migration revealed Tpm2.1-silenced cells exhibited a slower rate of wound closure compared to control cells, although they had large lamellipodia formed at the leading edge (Figure 2H, Supplementary Movie 1). Furthermore, EGF treatment of control cells showed decreased staining of E-cadherin between neighboring cells while Tpm2.1-silenced cells exhibited intact E-cadherin localization between neighboring cells. In addition, Tpm2.1-silenced cells exhibited increased stress fibers and large lamella at the leading edge (Figure 2I). These results indicate that downregulation of Tpm2.1 retards cell scatter in response to EGF.

### Downregulation of Tpm2.1 increases the rate of amoeboid and single cell migration and invasion

**Figure 3:**
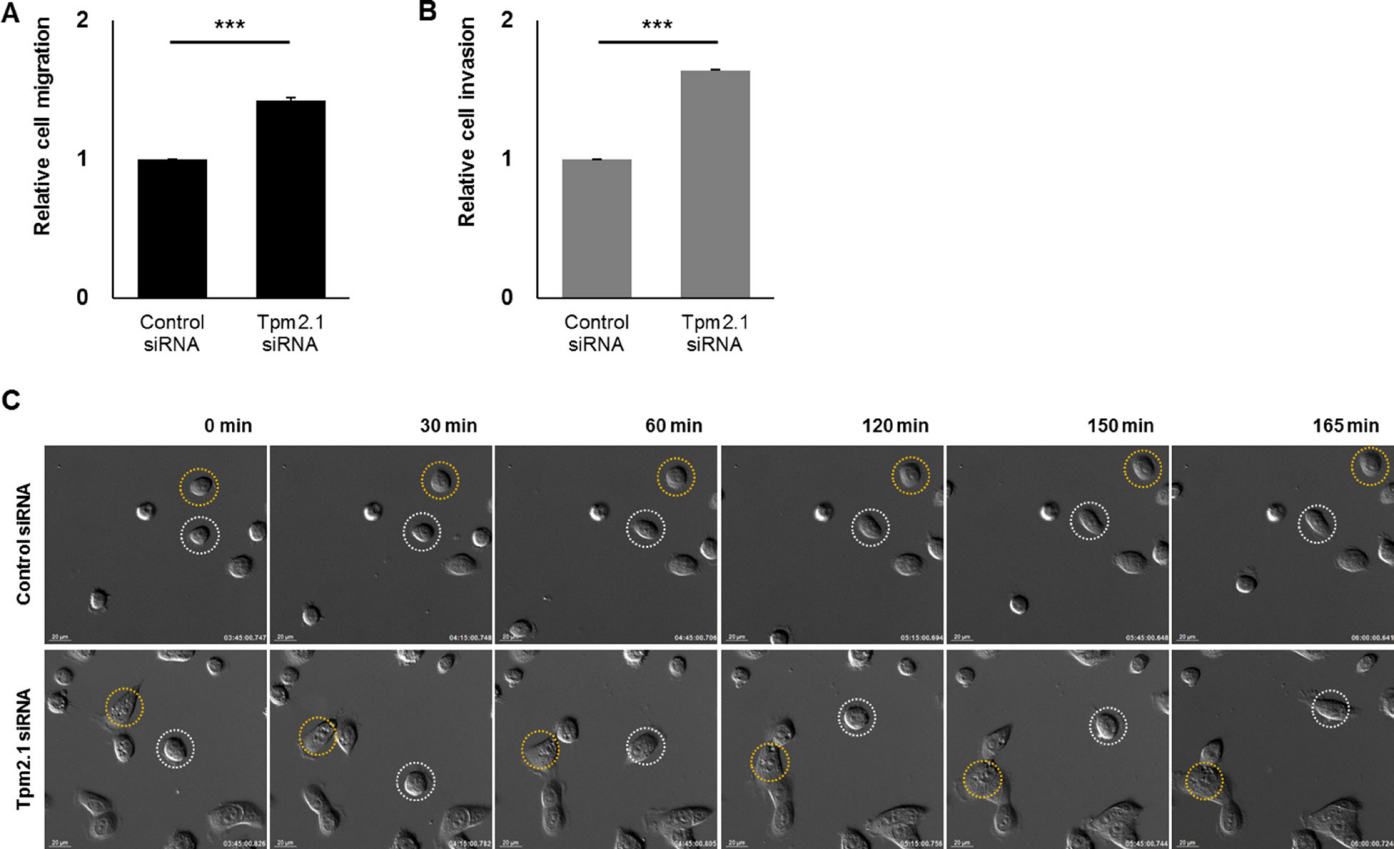
Downregulation of Tpm2.1 increases the rate of amoeboid cell migration, invasion and single cell migration (**A**–**B**) MCF10A cells were silenced with Tpm2.1 siRNA and were seeded on PET membranes to measure cell migration or Matrigel-coated membranes to measure invasion. The results represent four independent experiments (means ± s.e.m; ***P < 0.001; Student's *t*-test). (**C**) Live cell imaging was used to monitor single cell migration after RNAi treatment on fibronectin coated plates. White and yellow circles indicate cell motility in different time-lapse images (Scale bar: 20 μm).

We then analyzed the role of Tpm2.1 in amoeboid and mesenchymal or single cell migration. First we performed Boyden chamber assays. Tpm2.1 depletion in MCF10A cells resulted in increased migration through naked PET (polyethylene terephthalate) membrane (Figure 3A). To observe the invasiveness in Tpm2.1-silenced cells, membranes coated with Matrigel matrix were used. Tpm2.1-silenced cells showed an increase in invasion (Figure 3B). We next analyzed single cell migration on fibronectin using live cell imaging. Compared to the control cells, downregulation of Tpm2.1 resulted in a larger area of cell spreading on ECM and faster motility (Figure 3C, Supplementary Movie 2). Thus, in contrast of the results in the wound healing assays, downregulation of Tpm2.1 increased the rate of amoeboid and single cell migration and invasion.

### Tpm2.1 regulates the assembly of adhesion proteins in focal adhesions

**Figure 4:**
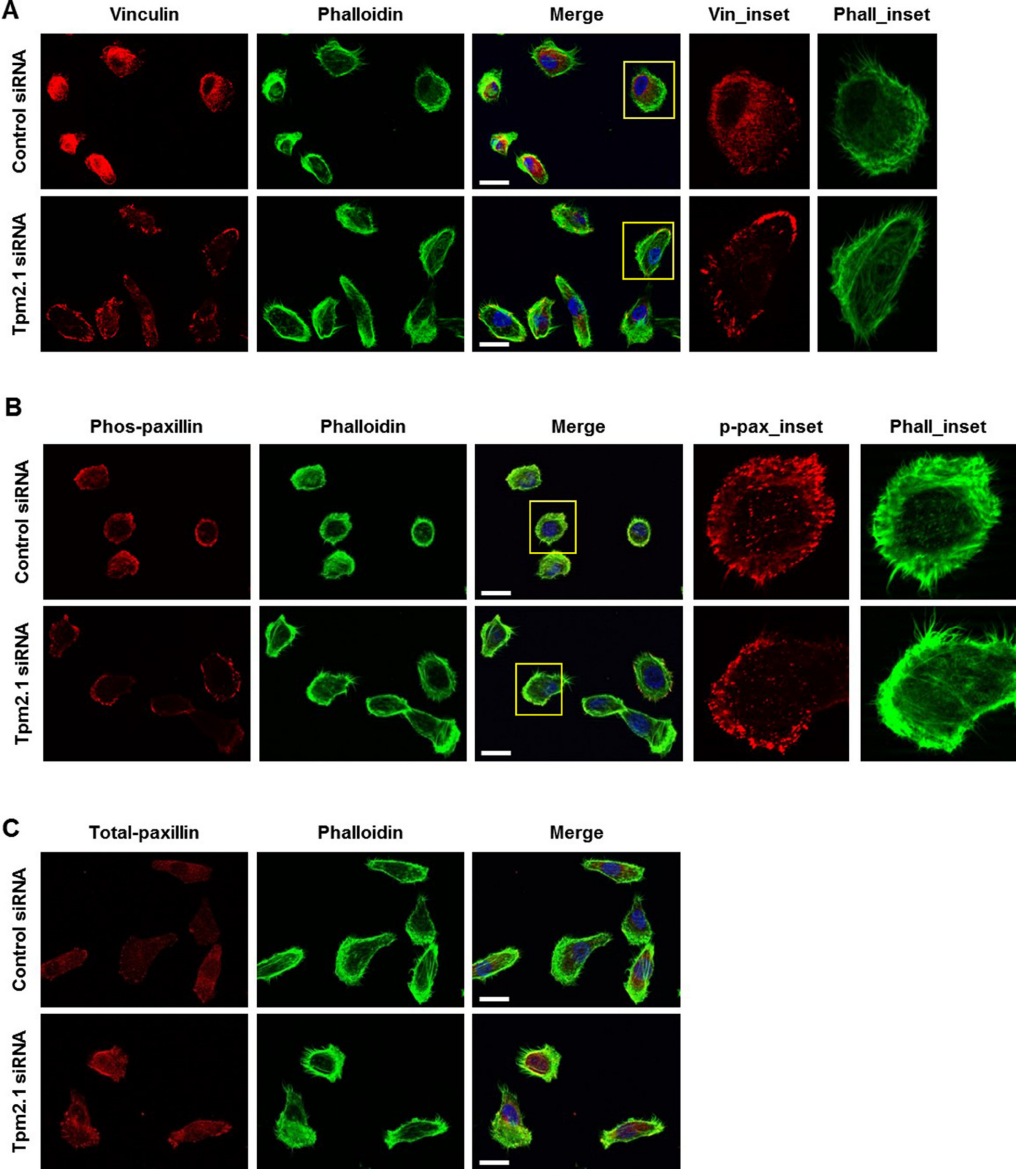
Tpm2.1 regulates the assembly of adhesion proteins in focal adhesion (**A**) Tpm2.1 siRNA-treated MCF10A cells adhered on fibronectin coated cover glass for 4 hours were stained for vinculin, actin filaments using phalloidin and DAPI (Scale bar: 20 μm). (**B**–**C**) Cells adhered for 4 hours, immunofluorescence against phospho-paxillin and total-paxillin (Scale bar: 20 μm). Yellow boxes were enlarged for detailed analysis (Inset).

Several reports suggest that focal adhesion complex proteins are mechanosensitive components that directly controls cell migration [9, 29, 30]. Additionally, there is evidence that Tpm2.1 plays an important role in mechanosensing [31]. To determine if Tpm2.1 plays a role in the assembly of adhesion complexes, single cells were stained for vinculin at different times after plating on fibronectin. After 4 hours on fibronectin, cells exhibited strong staining of vinculin at the cell edge and very little detected in the cell body in Tpm2.1 downregulated cells. By contrast, control cells exhibited weak vinculin staining at focal adhesions and more diffuse cytoplasmic staining (Figure 4A). We also examined the localization of another focal adhesion protein, paxillin and its phospho-form (site at Y118), which is important for adhesion complex assembly. Phospho-paxillin (Y118) was localized to the tip of the leading cell edge in silenced Tpm2.1 cells, while control cells showed localization at the edge and cytoplasm of the cell (Figure 4B). In addition, loss of Tpm2.1 increased total-paxillin localized to focal adhesions compared to the control (Figure 4C). Thus, depletion of Tpm2.1 increased the localization of focal adhesion proteins which might contribute to its effects on cell migration.

### Inhibition of Rho-kinase recovers motility during collective cell migration following downregulation of Tpm2.1

**Figure 5:**
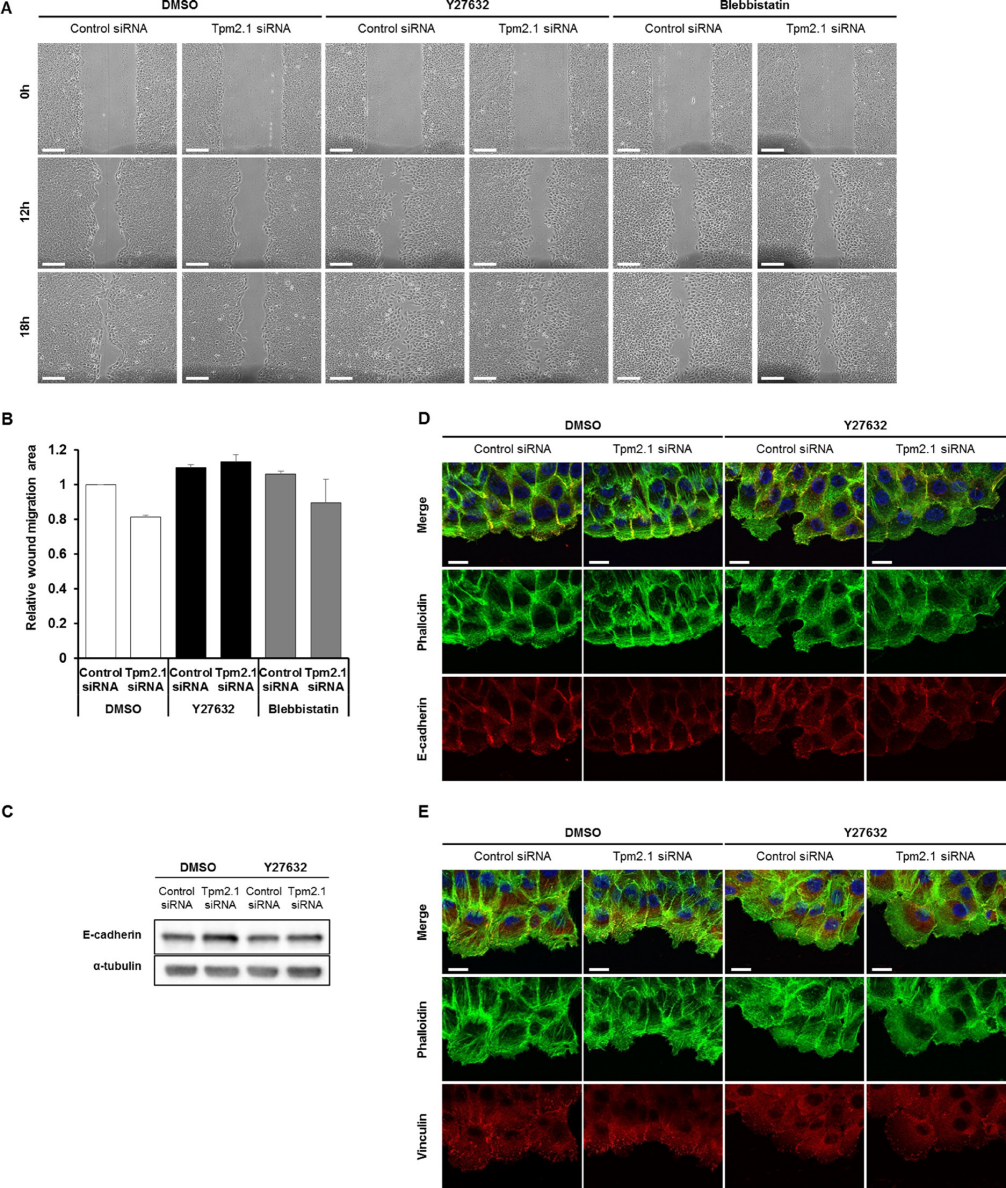
Inhibition of Rho kinase restores motility following downregulation of Tpm2.1 (**A**) Tpm2.1-silenced MCF10A cells were treated with 10 μM Y27632 or 25 μM blebbistatin and were scratched to make a wound (Scale bar: 100 μm). (**B**) Quantified data of A. More than three independent experiments were performed. (**C**) Immunoblotting data against E-cadherin of RNAi treated MCF10A cells followed by DMSO or Y27632 treatment. α-tubulin was used as a loading control. (**D**–**E**) The leading edge of cells undergoing wound healing were stained with antibodies against E-cadherin and vinculin, and actin filaments visualized with phalloidin and DAPI after Y27632 treatment of RNAi treated MCF10A cells. (Scale bar: 20 μm).

We next asked how downregulation of Tpm2.1 retards collective cell migration. A recent report showed that loss of Tpm2.1 increased actomyosin contractility [31]. Interestingly, studies by Yang and Kim reported that inhibition of ROCK with Y27632 in MCF7 cells led to loss of cell-cell adhesions and increased migration and invasion [32]. In addition, Cui *et al.* reported that loss of Tpm2.1 in colorectal cancer cell line HS675T upregulated the levels of active RhoA [33]. Based on these studies, we tested if inhibition of ROCK would reverse the effects of Tpm2.1-silencing during collective migration. MCF10A cells treated with siRNA or shRNA against Tpm2.1 recovered retarded cell migration after treatment with Y27632 (Supplementary Figure 2). Moreover, treatment of cells with blebbistatin partially restored collective cell migration (Figure 5A and 5B). Inhibition of ROCK and myosin II ATPase has been reported to impair E-cadherin-based adhesion [34]. In agreement with this report, we found that treatment of cells with Y27632 decreased actin filament formation at the edge of the wound where wider lamellipodia were formed and decreased localization of E-cadherin in cells at the leading edge (Figure 5D). We also observed upregulation of E-cadherin expression in Tpm2.1-silenced cells reversed following Y27632 treatment (Figure 5C). In addition, Y27632 treatment decreased the localization of vinculin at the leading edge and between neighboring cells in both control and Tpm2.1-silenced condition (Figure 5E). Hence, inhibition of ROCK using Y27632 rescued the retarded cell migration presumably by decreasing actomyosin contractility in Tpm2.1-silenced cells. Actomyosin contractility is essential for amoeboid cell migration [35, 36]. Consistent with this is the observation of increased amoeboid migration of Tpm2.1-silenced cells that was inhibited following Y27632 treatment (Supplementary Figure 3). Thus, Tpm2.1 modulates in actomyosin contractility mediated by Rho-ROCK that is essential in cell migration.

Rho GTPases play a critical role in cell migration as well as cell polarity. Rac and cdc42 are widely known to promote actin polymerization at the leading edge while Rho, plays a role in regulating actin assembly and actomyosin contractility at the trailing edge [11, 37]. HMW Tpms are localized at the basal membrane and participate at the leading edge of the migrating epithelial cells. We considered if Tpm2.1 could affect the localization of polarity proteins [6, 13]. We used a polarity marker GM130, a golgi marker, to stain cells undergoing collective cell migration. Our data showed that unlike the control cells where GM130 stained at a spot in front of the nucleus, Tpm2.1-silenced cells showed staining around the nucleus of the leading cells (Supplementary Figure 4). Therefore, we suggest that Tpm2.1 plays a role for well-organized cell polarity during collective cell migration.

### Downregulation of Tpm2.1 results in decreased level of AXL

**Figure 6:**
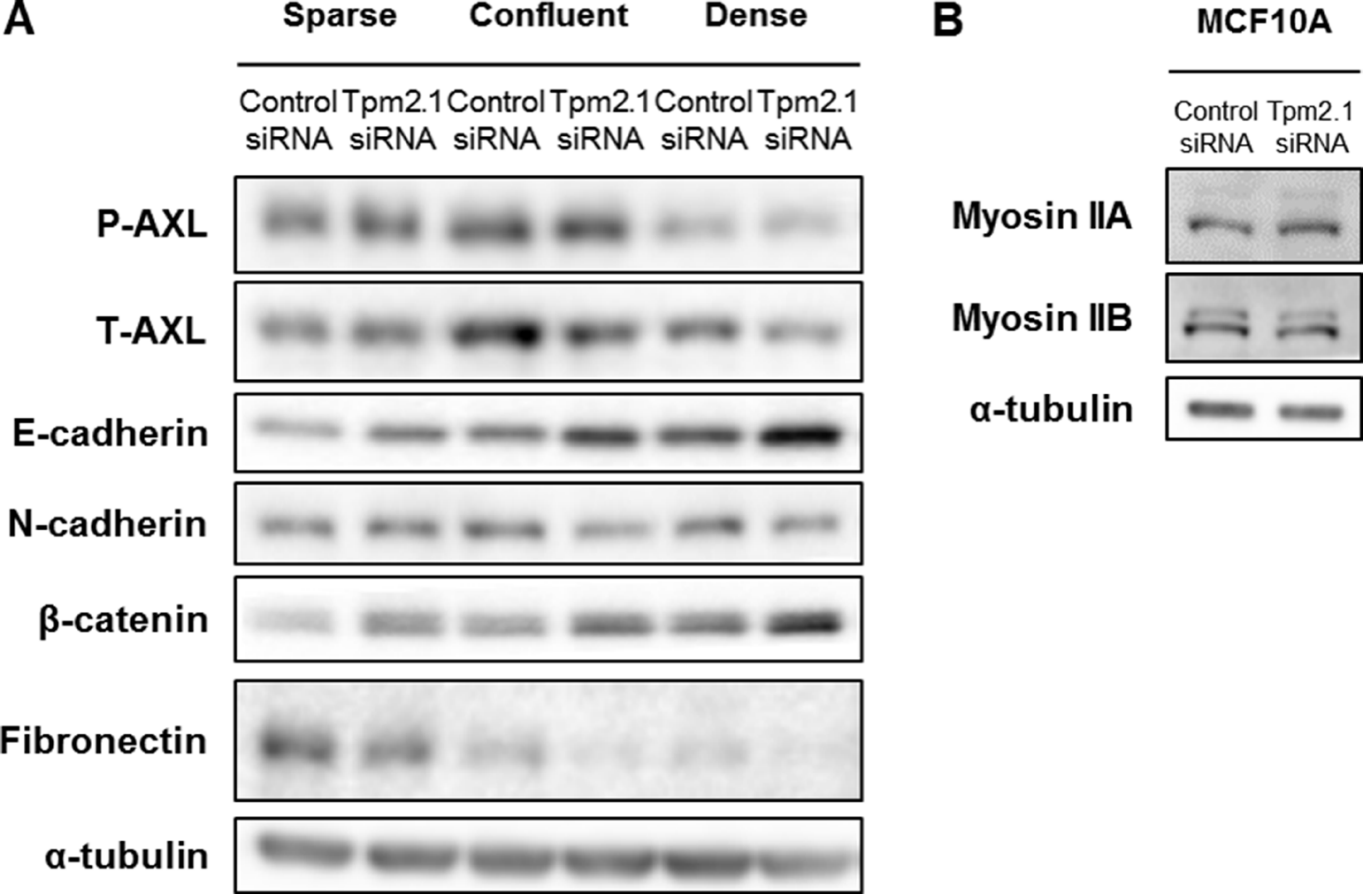
Downregulation of Tpm2.1 affects expression of AXL, E-cadherin and β-catenin in MCF10A cells (**A**) Cell lysates from MCF10A cells cultured under different densities were analyzed by immunoblot for expression of the indicated proteins. (**B**) Expression of myosin IIA and myosin IIB were analyzed by immunoblot. α-tubulin was used as a loading control.

Tpm2.1 is a binding partner of AXL receptor tyrosine kinase that also binds directly with myosin IIA [38]. AXL is a member of TAM family of receptor tyrosine kinases and a key regulator of EMT [39–41]. Activation of AXL affects EMT markers resulting in downregulation of E-cadherin and upregulation of N-cadherin or fibronectin [39, 40]. We hypothesized that Tpm2.1 might play a role in regulating cell migration via its effects on AXL signaling. We first compared the levels of AXL in cells grown under sparse, confluent and dense conditions. In confluent and dense condition cells, depletion of Tpm2.1 showed downregulation of AXL expression, even though the levels of phospho-AXL did not change (Figure 6A). Additionally, the levels of myosin IIA and IIB were no different between control and Tpm2.1-silenced cells (Figure 6B). To analyze the role of AXL downstream signaling in MCF10A cells, we observed that E-cadherin and β-catenin expression were upregulated while N-cadherin and fibronectin decreased. Under dense conditions E-cadherin and β-catenin increased the most, while levels of AXL are decreased (Figure 6A). Thus, downregulation of Tpm2.1 correlates with downregulation of AXL expression, and might be involved in upregulation of cell-cell adhesion proteins, E-cadherin and β-catenin.

### Loss of Tpm2.1 affects 3D epithelial cell aggregation and migration out from spheroids

**Figure 7:**
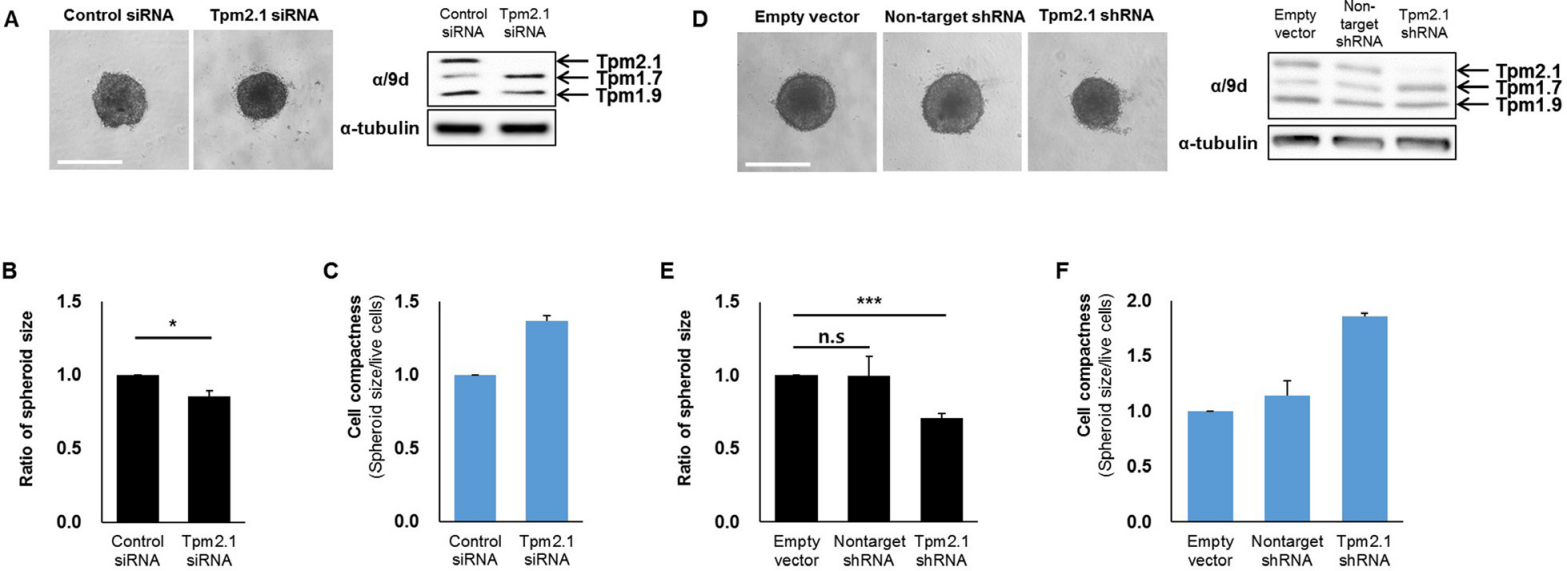
Downregulation of Tpm2.1 induces more compact spheroids (**A**, **D**) siRNA and shRNA transfected MCF10A cells were cultured in ultra-low attachment 96-well plates to form spheroids. After 96 hours, compact spheroids were formed and Tpms expression were detected by immunoblotting (Scale bar: 500 μm). (**B**, **E**) After 96 hours, quantification of the spheroid size were measured by SigmaScan Pro 4.0 and (**C**, **F**) Quantification of cell compactness were represented through cell viability by spheroid size. More than three independent experiments were performed (means ± s.e.m; *P < 0.05, ***P < 0.001; Student's *t*-test).

**Figure 8:**
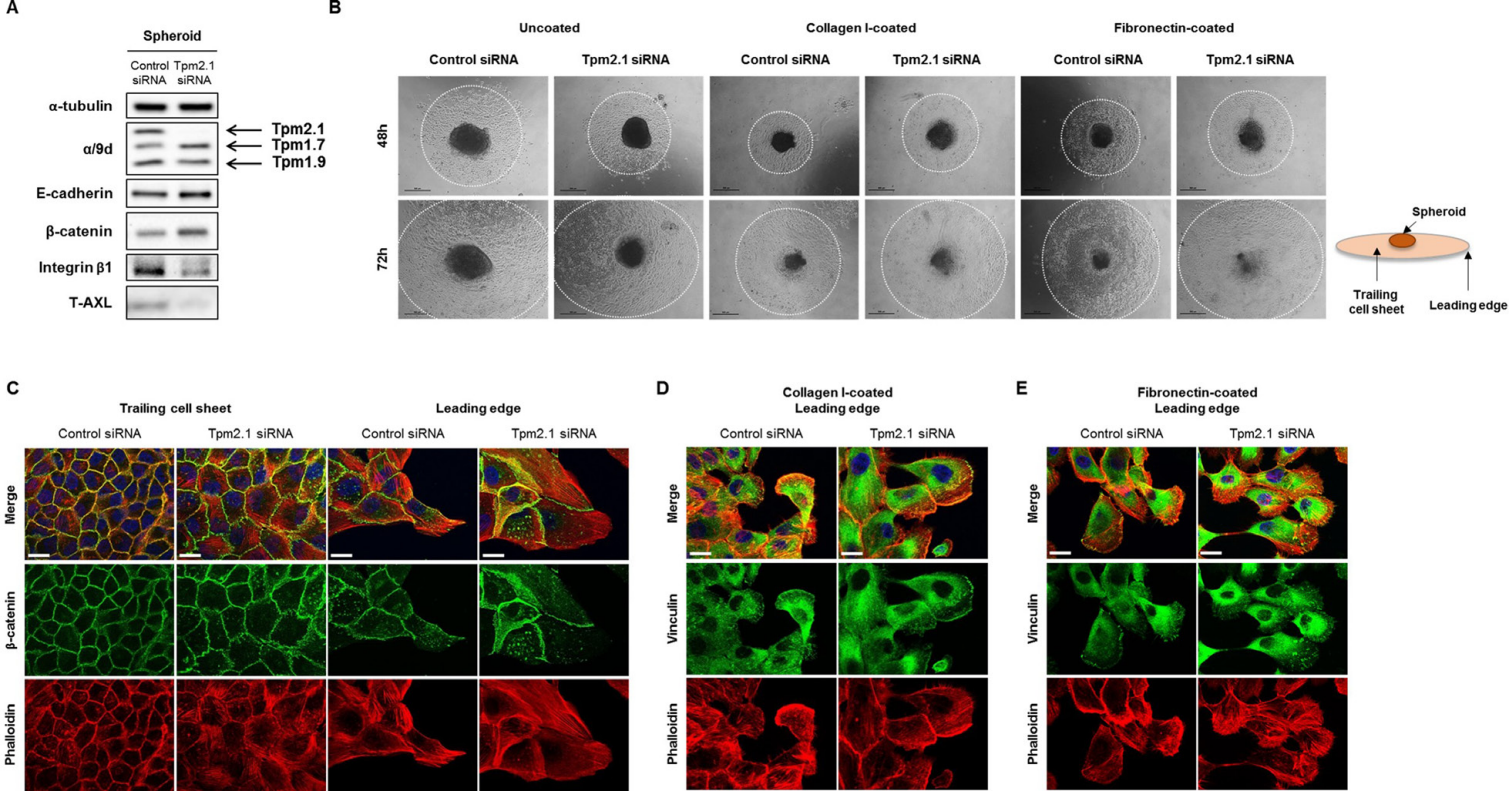
Rate of migration from spheroid depends on microenvironment (**A**) RNAi treated MCF10A cells were re-cultured in non-adherent plate and protein expression of total cell lysates was detected after 96 hours. (**B**) Spheroids were re-cultured on different substrates coated on culture plates to measure cells migrating out of the spheroids at 48 and 72 hours (Scale bar: 500 μm). (**C**) Immunofluorescent data for β-catenin, phalloidin and DAPI were imaged at the trailing cell sheet and leading edge of spheroid migration under uncoated culture condition. (**D**–**E**) Cells migrating out of the spheroid on collagen I and fibronectin were stained for vinculin, phalloidin and DAPI (Scale bar: 20 μm).

In addition to cell migration, cell aggregation is an important determinant in metastasis. Cell aggregation is thought to protect circulating cancer cells against immune cells. Additionally, survival and migration of aggregated cells into new extracellular environments is critical to metastasis [1, 42]. To test whether loss of Tpm2.1 affects cell aggregation, we analyzed the ability of cells to form cell spheroids. For these studies, we used shRNA against the Tpm2.1 gene as well as siRNA treated MCF10A cells. After 96 hours culture, Tpm2.1 siRNA or shRNA treated cells showed increased compaction of spheroids compared to the control cells (Figure 7A and 7D), and the size of spheroids were quantified (Figure 7B and 7E). Moreover, Tpm2.1 cells cultured on non-adherent plates exhibited an increase in cell compactness compared to control cells (Figure 7C and 7F). Next, we determined the expression of proteins in cells grown in spheroids that may contribute to cell aggregation. Immunoblot analyses showed increased levels of E-cadherin and β-catenin and decreased levels of integrin β1 and AXL in Tpm2.1-silenced cells compared to the control cells (Figure 8A).

We then analyzed the ability of cells migrating from a spheroid when spheroids were plated onto different matrices. Spheroids cultured for 72 hours, in non-adherent plates, were re-plated onto uncoated culture dishes or dishes coated with collagen I or fibronectin to observe cell migration which was measured at 48 and 72 hour time points (Figure 8B, white dotted circles). Compared to control cells, Tpm2.1-silenced spheroids showed retarded cell migration out of spheroids on to the uncoated culture plates. This result is similar to the results obtained for collective cell migration. Interestingly, when cells were introduced to plates coated with collagen I or fibronectin, Tpm2.1-silenced cells migrated faster away from the spheroids compared to control cells (Figure 8B). Cells migrating away from the spheroids were fixed and stained for actin, β-catenin and vinculin. At the trailing cell sheet, β-catenin expression co-localized with cortical actin filaments in control cells while Tpm2.1-silenced cells showed a disrupted actin arrangement around the cortical region. At the leading edge, control cells showed short actin filaments around the cell cortex, whereas Tpm2.1-silenced cells exhibited increased stress fibers across the cell and at the cortical region in Tpm2.1-silenced cells. At the trailing cell sheet, β-catenin showed no difference in both control and Tpm2.1-silenced cells, but was increased in cell-cell adhesions at the leading edge of Tpm2.1-silenced cells (Figure 8C). Interestingly, cells from the spheroids exposed to collagen I and fibronectin showed similar actin arrangements and vinculin localization at the cell edge between the control and Tpm2.1-silenced cells (Figure 8D and 8E). In conclusion, loss of Tpm2.1 increases the ability of breast epithelial cells to aggregate and exposure to different ECM microenvironment increases cell migration out of spheroids, which may play a role during metastasis.

## DISCUSSION

Decreased expression of specific high molecular weight isoforms of tropomyosin is a common feature of transformed and tumor cells which has been implicated in the abnormal properties of cancer cells [15, 17, 19, 21, 22, 43]. Here, we studied the role of Tpm2.1 in non-transformed MCF10A cells and the effects of downregulation of this isoform on cell morphology, cytoskeletal organization, migration and aggregation. Based on the experiments presented here, we suggest a role for downregulation of Tpm2.1 in altered cell migration and invasion, thereby contributing to cancer progression and metastasis.

### Function of Tpm2.1 in collective cell migration

Downregulation of Tpm2.1 inhibited collective cell migration of cells plated on uncoated culture plates, or culture plates coated with either fibronectin or collagen. Because a number of studies have reported that ectopic expression of Tpm1.7 can inhibit the migration of transformed epithelial cells, we first hypothesized that expression of Tpm1.7 might be responsible for the slower rate of migration following downregulation of Tpm2.1 [16]. However, silencing both Tpm1.7 and Tpm2.1 did not enhance the rate of collective cell migration (Figure 2B and 2C).

Collective cell migration requires a decrease in actomyosin contractility at cell-cell contacts involving a decrease in Rho-ROCK signaling [44]. Interestingly, loss of Tpm2.1 has been found to increase Rho-GTP levels and activation of Rho-ROCK mediated regulation of actomyosin contraction [33]. Consistent with these previous studies, we found that retardation of collective cell migration by loss of Tpm2.1 was reversed by inhibition of ROCK (Figure 5). In addition, we found loss of Tpm2.1 disrupted cell polarity at the leading edge (Supplementary Figure 4). These results suggest that Tpm2.1 regulates actomyosin contractility via Rho-signaling and together with cell polarity regulators is important for collective cell migration.

### Loss of Tpm2.1 increased amoeboid and single cell migration

In contrast to the effects on collective cell migration, Tpm2.1 depletion using siRNA resulted in increased amoeboid migration and invasion, which was inhibited by the ROCK inhibitor Y27632 (Figure 3A and 3B, Supplementary Figure 3). The increase in amoeboid migration may be due to the action of active Rho-ROCK signaling, which is important for amoeboid movement [45]. Hence, we could conclude that increase in Rho signaling and actomyosin contractility following downregulation of Tpm2.1 enhances the rate of amoeboid migration.

The localization of focal adhesion proteins were also affected in different modes of cell migration. Tpm2.1 is reported to be localized at the cell edge with the focal adhesion proteins [6, 9]. One study showed that vinculin-paxillin interaction was induced by myosin II mediated FAK phosphorylation of paxillin, which recruited actin for activation of tension-mediated focal adhesion maturation [46]. We suggest that an increase in actomyosin contractility may explain the stabilization and increased localization of focal adhesion proteins in single Tpm2.1-silenced cells. On the other hand, because of the strong adhesion between the neighboring cells during collective migration the force generated by the focal adhesion would be relatively weak contributing to slower collective migration in Tpm2.1-silenced cells.

### Role of Tpm2.1 in cell aggregation and migration from spheroids

Recent studies show that clusters of cancer cells were more invasive than single tumor cells. Tumor cells in clusters were more resistant to apoptosis and exited faster from the bloodstream to a distant organ [47]. More compact spheroids were observed in Tpm2.1-silenced MCF10A cells compared to control cells (Figure 7). Spheroid formation initially involves ECM-integrin interaction of dispersed single cells, and then a delay period of E-cadherin proteins accumulation, followed by strong homophillic interaction of E-cadherins [48]. Our data suggests that upregulation of E-cadherin and β-catenin following Tpm2.1-silencing may play an important role in cell aggregation by increasing strong cell cohesion, leading to more compact cell clusters. Cell fractionation assays showed significant increase of β-catenin in the membrane and cytoskeletal matrix in Tpm2.1-silenced cells. Interestingly, we observed a slight increase in β-catenin in the nucleus after depletion of Tpm2.1 (Supplementary Figure 1). It remains to be determined if decreased expression of Tpm2.1 may activate β-catenin transcriptional signaling.

Tpm2.1-silenced cells migrated out of the spheroids more efficiently than control cells depending on the ECM matrices. A key determinant of metastasis is the microenvironment of the distant organ [42]. Recent evidence suggests that the main trigger for cells to proliferate and become invasive are the changes in the ECM. Initially, tumor cells prepare the metastatic microenvironment as they have the ability to secrete factors for their survival and proliferation [2, 11, 49]. Fibronectin is a tumor cell-derived ECM component necessary for metastatic spread [11]. Increased levels of fibronectin can establish a pre-metastatic niche. Hence, fibronectin may act as a signal recognized by the circulating cancer cells, playing a role in cancer metastasis [50]. In addition, tumorigenesis results in dramatic changes in cells including EMT. Exposure of MCF10A cells to fibronectin activated mesenchymal markers such as snail, N-cadherin, vimentin as well as fibronectin itself [51]. Our data showed that when MCF10A cells were exposed to fibronectin, unlike culture plate, the level of fibronectin increased in both control and Tpm2.1-silenced MCF10A cells (Supplementary Figure 5). Thus, although both control and Tpm2.1 silenced cells exhibit a positive feedback expression of fibronectin secretion, Tpm2.1-silenced cells migrated faster on differently coated ECMs. It still remains to be determined why Tpm2.1-silenced cells migrated faster out of the spheroids.

Collectively these data show that non-muscle Tpm2.1 plays a role in the regulation of cell migration and cell aggregation. Moreover, downregulation of Tpm2.1 increased the invasive properties of cell aggregates when they encounter a new microenvironment. These studies provide new insights into the role of Tpm2.1 in normal cell function and how loss of this isoform can contribute to cancer invasion and metastasis.

## MATERIALS AND METHODS

### Reagents and antibodies

Reagents. DMSO (Sigma-Aldrich, D8418), Y27632 (Enzo Life Science, ALX-270-333), Blebbistatin (Enzo Life Science, EI-315-0005).

Antibodies. The following antibodies were used: α9d (a gift from Peter W Gunning), α-tubulin (Sigma-Aldrich, T5168), E-cadherin (BD Biosciences, 610181), and vinculin (Sigma-Aldrich, V9131); AXL (H-3) (Santa Cruz Biotechnology, sc-166269), p-AXL (Y779) (R&D systems, AF2228), β-catenin (BD Biosciences, 610154), and Integrin β1 (Cell Signaling Technology, 4706); N-cadherin (Abcam, ab18203), Fibronectin (BD Biosciences, 610077), p-paxillin (Y118) (Cell Signaling Technology, 2541) and paxillin (Santa Cruz Biotechnology, sc-5574); Myosin IIA (Covance, PRB-440P), Myosin IIB (Covance, PRB-445P), and GM130 (BD Biosciences, 610822).

### Cell culture

MCF10A non-malignant breast epithelial cells (American Type Culture Collection (ATCC)) were cultured in Dulbecco's modified Eagle's medium/F12 (Welgene) supplemented with 5% horse serum (Gibco), 10 μg/ml bovine insulin (Roche), 0.5 μg/ml hydrocortisone (Sigma-Aldrich), 0.1 μg/ml cholera toxin (Sigma-Aldrich), 20 ng/ml epidermal growth factor and 1% penicillin/streptomycin (Welgene). Breast cancer cell lines (MCF7, T47D, BT-474, SK-BR-3, BT-20, MDA-MB-231, and MDA-MB-468) and HeLa cell were purchased from ATCC and cultured according to the manufacturer's instructions. Cells were incubated at 37°C in humidified 5% carbon dioxide cell incubator.

### siRNA and shRNA transfection

For siRNA transfection, cells (2.0 × 10^5^ cells per dish) were plated on 60 mm dish for 1 day, and then transfected with 50 nM of siRNAs against human TPM2 (gene ID 22004) using two different siRNAs (Thermo Scientific and Bioneer), and human scrambled negative control siRNAs (Bioneer) for control using Lipofectamine RNAiMAX reagent (Invitrogen) following the manufacturer's protocol. After 72 hours of transfection, cells were detached by using trypsin-free cell detachment solution, HyQtase (Thermo Scientific) and were applied to required experiments.

For shRNA (Sigma-Aldrich) transfection, MCF10A cells were subsequently infected with retroviral vectors encoding for TPM2 and selected.

### Immunoblotting

Cells were lysed in ice-cold 2X laemmli sample buffer (0.125 M Tris-HCl, 20% glycerol, 4% sodium dodecyl sulfate and 0.004% Bromophenol blue) with additional protease inhibitor cocktail (Sigma-Aldrich) and phosphatase inhibitors cocktail (Roche). The total protein extracts were separated by SDS-PAGE and transferred to a 0.2 μm nitrocellulose membrane. Membrane was probed with primary antibody for 1 hour at room temperature or overnight incubation at 4°C followed by incubation in peroxidase-conjugated AffiniPure goat anti-mouse IgG and goat anti-rabbit IgG (Jackson ImmunoResearch Laboratories) at 1:2000 dilution. To detect proteins of interest, peroxidase substrate was applied for enhanced chemiluminescence.

### Wound healing migration assay

MCF10A Cells were seeded on either culture plate, 0.001% fibronectin- (Sigma-Aldrich) or 0.01% collagen I- (Sigma-Aldrich) coated 6 well plate for 2 days. Confluent cells were then wounded with 200 μl plastic tips across the cell monolayer. After scraping the cell monolayer, fresh complete culture medium of the cell line was added. Images were taken by phase-contrast microscope (Eclipse TS100, Nikon). The area of wound closure was quantitated using Image J software (NIH). For live cell imaging, RNAi-treated cells were incubated on glass plate at 37°C with 5% carbon dioxide humidify chamber of inverted ZEISS microscope. DIC time-lapse images were collected and were made as a video using AxioVision 4.8 AutoMeasure module.

### Indirect Immunofluorescence

Cells were plated on uncoated or fibronectin-coated glass coverslips in 6 well plates. At indicated time, cells were co-fixed with 0.1% Triton-X-100 and 4% formaldehyde for 2 min, and then with 4% formaldehyde for another 15 min. Cells were probed with primary antibodies for 30–45 min, followed by incubation of secondary antibodies (Invitrogen) mixed with phalloidin conjugated with Oregon green or Alexa 594 (Invitrogen) for 30 min at room temperature. Before mounting, 4,6-Diamidino-2-phenylindole (DAPI) (1 μg/ml) diluted in 1× PBS was incubated for 5 min. Prolong Gold antifade reagent (Invitrogen) was used for mounting coverslips. Cell fluorescence was observed with ZEISS Observer Z1 microscope with Apotome 2. Image acquisition and processing were performed with AxioVision 4.8.

### Transwell migration and invasion assay

Cells (1.0 × 10^5^ cells/well) grown in serum-free medium for 24 hours were seeded on upper chamber of 8.0 μm pore size transparent PET membrane or Matrigel-coated invasion chamber (BD Bioscience). After 48 hours membrane was removed and fixed with 4% formaldehyde (Sigma-Aldrich) to quantify cell migration to the lower chamber induced by serum in cell medium. Nucleus of migrated cells were stained with DAPI and detected using ZEISS AxioVision 4.8 AutoMeasure module (Zeiss). Five fields on each membrane was averaged to quantify the level of cell migration. Each condition was duplicated in an independent experiment.

### Single cell migration and cell scatter assay

RNAi-transfected MCF10A were used to analyze single cell migration assay. Detached cells with HyQtase were resuspended with MCF10A complete media. After centrifugation, 1.0 × 10^5^ cells were diluted in complete medium, and adhered on 0.001% fibronectin-coated plate and incubated at 37°C. After 2 hours incubation, unattached cells were washed out, and motility of single cells were monitored. Single cell motility was captured by DIC time-lapse microscopy for every 2 min until 6 hours. For cell scatter assay, MCF10A cells were seeded at a density of 5.0 × 10^4^ per sample for 24 hours. For another 24 hours, cells were serum and growth factor starved to induce aggregate formation. Cell aggregates were then stimulated with 100 ng/ml EGF and imaged by DIC time-lapse microscopy for 8 hours.

### Spheroid formation and migration assay

Cells were cultured in 96-well ultra-low attachment round bottom plate (Corning) at a concentration of 5000 cells per well. The spheroids were allowed to grow for 72–144 hours at 37°C. Sizes of formed spheroid after 72 hours were measured using SigmaScan Pro 4.0. Then the spheroids were transferred to culture plate, 0.001% fibronectin- or 0.01% collagen I- coated plate and maintained for 48 hours before detection.

### Statistical analysis

For all parametric data, an unpaired, two-tailed Student's *t*-test was used to determine significance. Data were analyzed with Microsoft Excel.

## SUPPLEMENTARY MATERIALS FIGURES










