# Supplementary material for: Tropomyosin isoform Tpm2.1 regulates collective and amoeboid cell migration and cell aggregation in breast epithelial cells
Source: Oncotarget. 2017 Jul 12;8(56):95192–205. doi: 10.18632/oncotarget.19182 (PMC5707015; doi:10.18632/oncotarget.19182)
Supplement: Supplementary file 1 [file oncotarget-08-95192-s001.pdf]

Tropomyosin isoform Tpm2.1 regulates collective and amoeboid cell migration and cell aggregation in breast epithelial cells

SUPPLEMENTARY MATERIALS

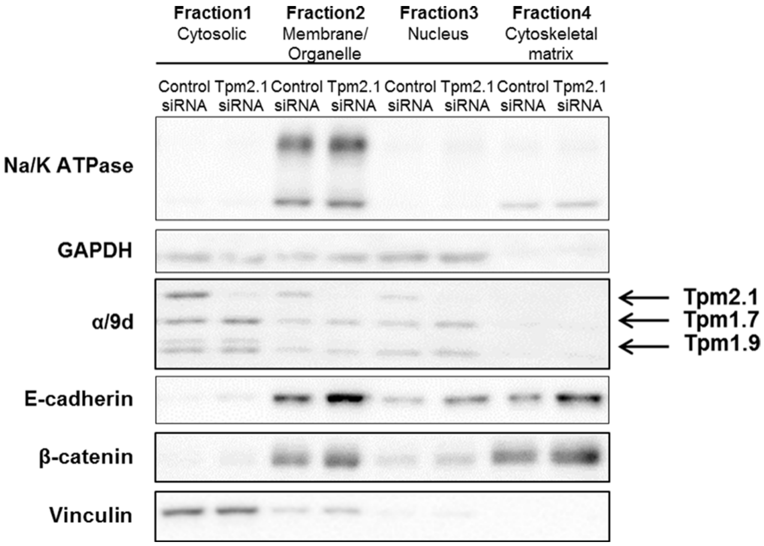

Supplementary Figure 1: Downregulation of Tpm2.1 affects the level of cell-cell adhesion proteins in the membrane. Protein expression of cell fractionated MCF10A cells were detected. Na/K ATPase and GAPDH was detected for fraction marker.

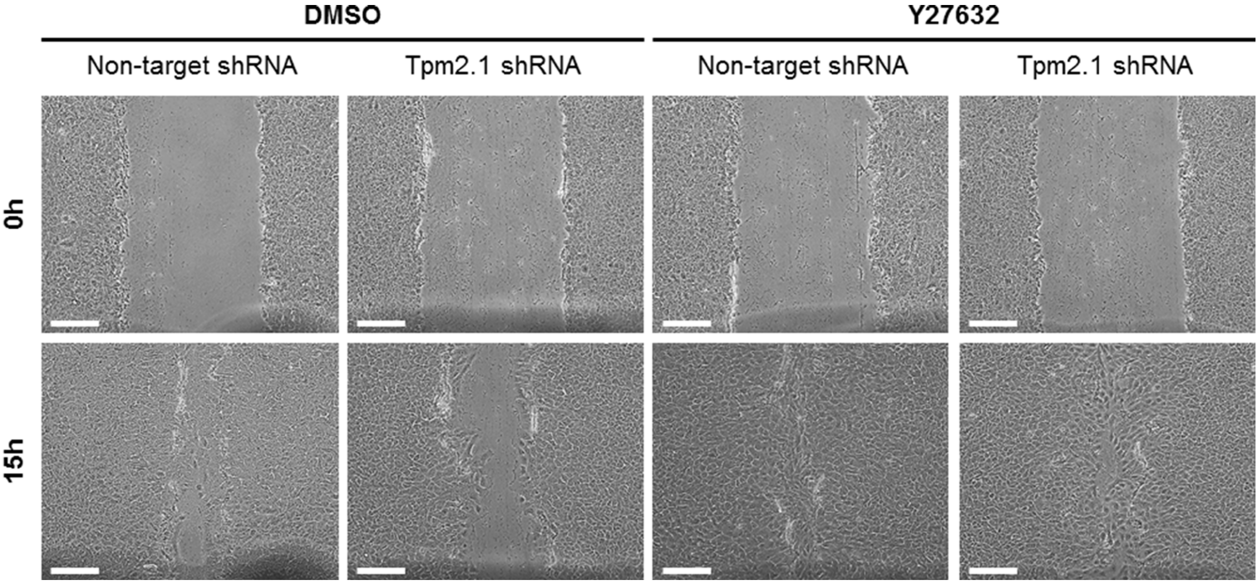

Supplementary Figure 2: Inhibition of Rho kinase recovered collective migration following downregulation of Tpm2.1. shRNA- treated against Tpm2.1 in MCF10A cells were treated with 10 μM Y27632 and were scratched to make a wound (Scale bar: 100 μm).

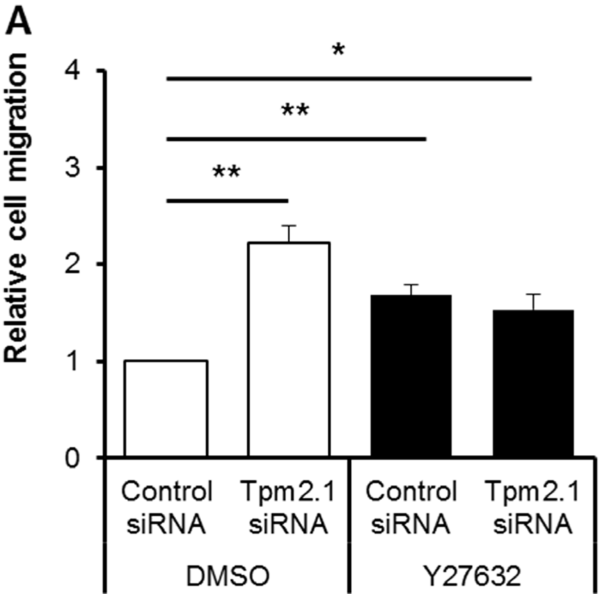

Supplementary Figure 3: Inhibition of Rho kinase affects the rate of amoeboid cell migration and invasion. (**A–B**) MCF10A cells were silenced with Tpm2.1 siRNA and were seeded on PET membrane to detect cell migration (A), or Matrigel-coated membrane for invasion (B).

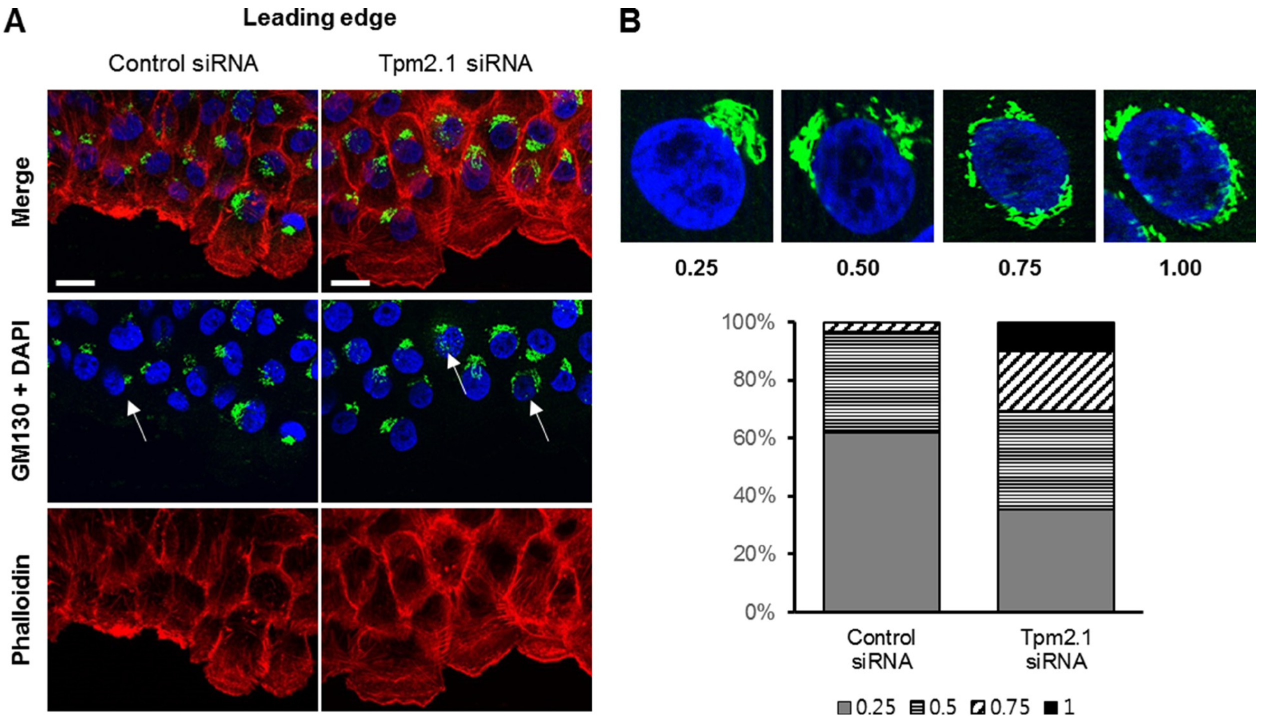

Supplementary Figure 4: Tpm2.1 silencing affect cell polarity. (**A**) The leading edge of RNAi treated MCF10A cells undergoing wound healing were stained with antibodies against GM130, phalloidin and DAPI. (Scale bar: 20 µm) (**B**) Expression of GM130 was quantified by counting the expression at the leading edge of the wound, categorizing the cells with a reference shown of GM130 expression area around the nucleus (*n* = 60).

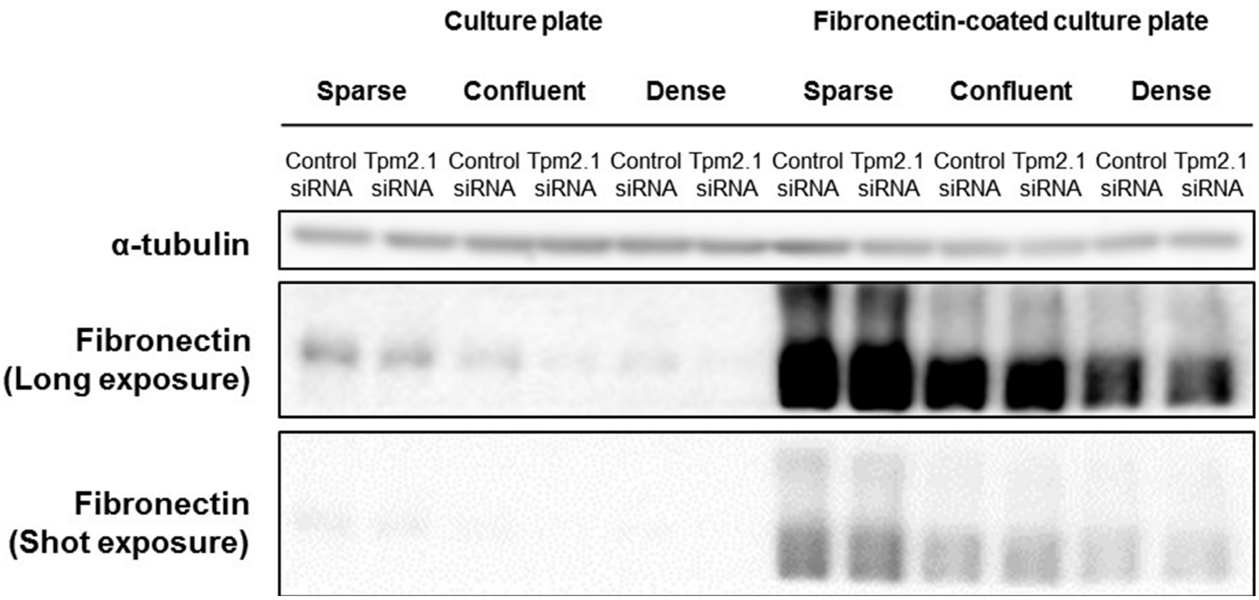

Supplementary Figure 5: Exposure to fibronectin induces positive feedback in MCF10A cells. RNAi- treated against MCF10A cells were seeded on culture plate or fibronectin-coated culture plate under different cell density and total cell lysates were detected against fibronectin. α-tubulin was used as a loading control.

Supplementary Movie 1: EGF-activated wound healing migration in MCF10A cells. 100 ng/ml EGF treated (**A**) control and (**B**) Tpm2.1-silenced MCF10A cells were wounded to moniter EGF activation during wound healing. See Supplementary_Movie_1

Supplementary Movie 2: Downregulation of Tpm2.1 increases single cell migration. (**A**) Control and (**B**) Tpm2.1-silenced MCF10A cells were cultured on fibronectin coated plates to monitor single cell migration. See Supplementary_Movie_2
